# Changes in central retinal artery blood flow after ocular warming and cooling in healthy subjects

**DOI:** 10.4103/0301-4738.62641

**Published:** 2010

**Authors:** M A Shamshad, A K Amitava, I Ahmad, S Wahab

**Affiliations:** Institute of Ophthalmology, JNMC, AMU, Aligarh, India; 1 Department of Radiodiagnosis, JNMC, AMU, Aligarh, India

**Keywords:** Central retinal arteries, end diastolic velocity, peak systolic velocity, temperature

## Abstract

**Context:**

Retinal perfusion variability impacts ocular disease and physiology.

**Aim:**

To evaluate the response of central retinal artery (CRA) blood flow to temperature alterations in 20 healthy volunteers.

**Setting and Design:**

Non-interventional experimental human study.

**Materials and Methods:**

Baseline data recorded: Ocular surface temperature (OST) in °C (thermo-anemometer), CRA peak systolic velocity (PSV) and end diastolic velocity (EDV) in cm/s using Color Doppler. Ocular laterality and temperature alteration (warming by electric lamp/cooling by ice-gel pack) were randomly assigned. Primary outcomes recorded were: OST and intraocular pressure (IOP) immediately after warming or cooling and ten minutes later; CRA-PSV and EDV at three, six and nine minutes warming or cooling.

**Statistical Analysis:**

Repeated measures ANOVA.

**Results:**

(n = 20; μ ± SD): Pre-warming values were; OST: 34.5 ± 1.02°C, CRA-PSV: 9.3 ± 2.33 cm/s, CRA-EDV: 4.6 ± 1.27 cm/s. OST significantly increased by 1.96°C (95% CI: 1.54 to 2.37) after warming, but returned to baseline ten minutes later. Only at three minutes, the PSV significantly rose by 1.21 cm/s (95% CI: 0.51to1.91). Pre-cooling values were: OST: 34.5 ± 0.96°C, CRA-PSV: 9.7 ± 2.45 cm/s, CRA-EDV: 4.7 ± 1.12 cm/s. OST significantly decreased by 2.81°C (95% CI: −2.30 to −3.37) after cooling, and returned to baseline at ten minutes. There was a significant drop in CRA-PSV by 1.10cm/s (95% CI: −2.05 to −0.15) and CRA-EDV by 0.81 (95% CI: −1.47 to −0.14) at three minutes. At six minutes both PSV (95% CI: −1.38 to −0.03) and EDV (95% CI: −1.26 to −0.02) were significantly lower. All values at ten minutes were comparable to baseline. The IOP showed insignificant alteration on warming (95% CI of difference: −0.17 to 1.57mmHg), but was significantly lower after cooling (95% CI: −2.95 to −4.30mmHg). After ten minutes, IOP had returned to baseline.

**Conclusion:**

This study confirms that CRA flow significantly increases on warming and decreases on cooling, the latter despite a significant lowering of IOP.

The ocular circulation is geared to meet the nutritional needs and respond to physiological changes to allow the specialized tissues to function optimally.[1] Understanding ocular blood flow changes in response to temperature alterations is of particular importance since these modalities in the form of transpupillary thermotherapy and cryo-applications are being increasingly used. This is possible with increasing availability of ultrasound-based Color Doppler Imaging (CDI) to evaluate blood flow velocities in the ophthalmic, central retinal and posterior ciliary arteries.[2] Recently, color Doppler optical coherence tomography,[3] Heidelberg retinal flowmetery[4] and a stabilized retinal laser Doppler instrument[5] have also been used for imaging the ocular vascular dynamics.

Ocular warming in healthy individuals increases the retinal blood flow (RBF), while decreasing the choroidal blood flow (CBF).[6] The decrease in CBF is thought to prevent retinal (especially macular) damage consequent to ocular hyperthermia.[7] Since eyes may experience high temperatures both as an occupational hazard and therapy, it is important to evaluate ocular blood flow alterations in response to ocular warming.

Chilling the eye is often resorted to, as in cyclocryotherapy and retinal cryopexy. People at high altitude, polar explorers or those exposed to snowstorms, avalanches or freezing waters risk cryo-injury. No studies were found evaluating the effect of cooling on ocular blood flow (MEDLINE). Katsimpris demonstrated in rabbits that trans-palpebral ocular cooling significantly lowered aqueous and vitreous temperatures, comparable to the effect of direct corneal chilling.[8]

Utilizing CDI, we studied the changes in central retinal artery (CRA) blood flow on warming and cooling normal eyes.

## Materials and Methods

The study was approved by the institutional review board of the Institute of Ophthalmology, Aligarh Muslim University and informed consent was taken. Twenty healthy young volunteers were recruited from amongst the junior residents of the ophthalmology department. The inclusion criteria were best corrected visual acuity (BCVA) of 20/20 Snellen, with normal anterior segment on slit-lamp biomicroscopy and a normal fundus on indirect ophthlamoscopy. Any volunteer with fever, history suggestive of a rheological disorder, such as diabetes or hypertension, glaucoma, maculopathy, pathological myopia or tear film abnormalities (on Schirmer testing), subjects who had undergone any ocular surgery or were contact lens wearers were excluded. A carefully supervised pilot study was done on five volunteers, where warmth and cooling was done for gradually increasing periods (up to ten minutes) and VA and biomicrosopy carried out at each interval to assess any adverse effect of the intervention.

**Figure 1:**
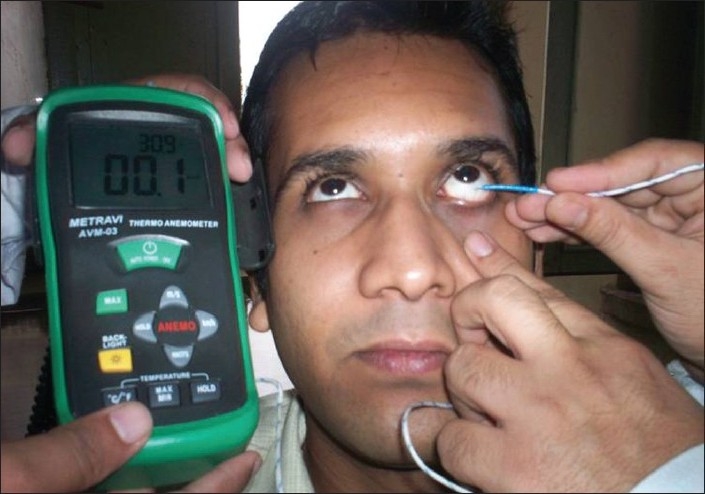
The thermo-anemometer with the probe in contact with the bulbar conjunctiva in a study subject

Subjects abstained from drinking tea, coffee and smoking two hours prior to the test and rested for ten minutes before the tests. The study was performed in an air-conditioned room with a controlled temperature range of 20°C-24°C, humidity range of 20-25%, and constant brightness. Baseline measures recorded were: Ocular surface temperature (OST) in °C, using a thermo-anemometer (Metravi AVM-03, Arun Enterprises, Kolkata) with resolution of 0.1°C, range of −20 to 200°C and an accuracy of ±0.8°C. The probe was placed in contact with the lower bulbar conjunctiva [Fig. 1] and the temperature read off after allowing 30 sec for stabilization.

**Figure 2:**
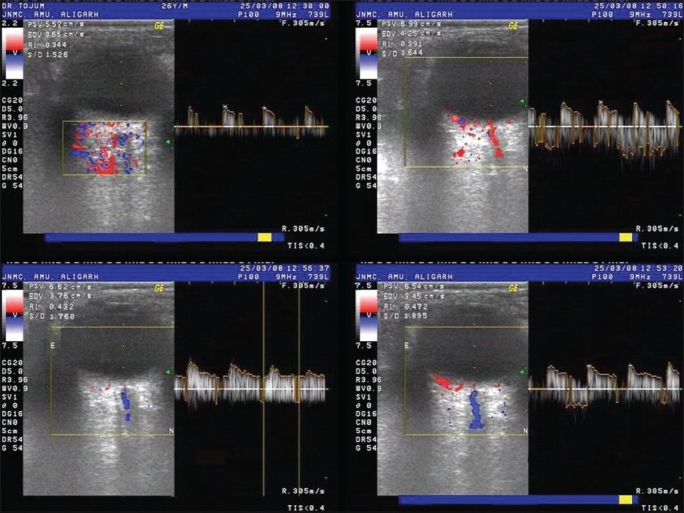
Clockwise from top left: Color Doppler images of central retinal artery blood flow at baseline and at 3, 6 and 9 min after warming

**Figure 3:**
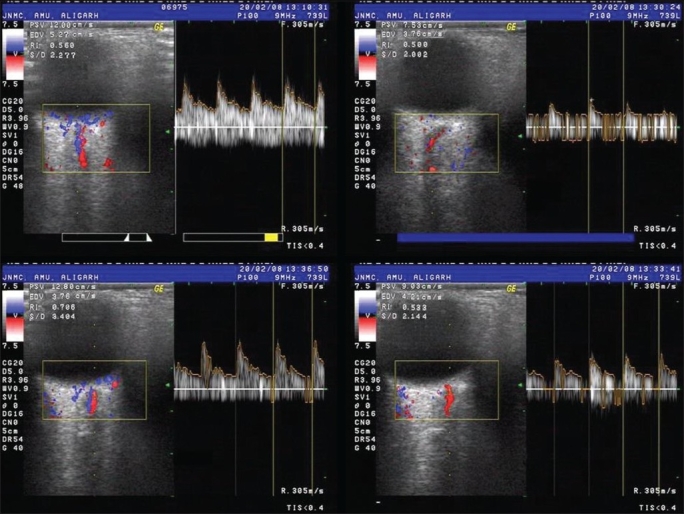
Clockwise from top left: Color Doppler images of central retinal artery blood flow at baseline and at 3, 6 and 9 min after chilling

CRA velocity (cm/sec) was measured by Color Doppler images [Figs. 2 and 3] obtained by using the GE Logiq 500 PRO series Color Doppler Ultrasound device (GE Healthcare, U.K.). The sonographer was unaware whether the eye had been warmed or cooled. Using image superimposition the central retinal vessels were identified and with the sample volume (pulse length 1.5 mm) centered three mm behind the optic disc surface, quality flow-velocity verses time curves were obtained. The peak systolic velocity (PSV) and the end diastolic velocity (EDV) of CRA were recorded. The resistive index (RI) was calculated: RI = (PSV − EDV)/PSV.

The laterality of the eyes (right or left) and the temperature alteration (warming or cooling) were randomly assigned by the toss of a coin. Between temperature alterations each subject was allowed a ten-minute break. For ocular warming an electric lamp having one 60 watt tungsten bulb was placed ten cm from the closed lids for ten minutes. The cooling was achieved by using an ice-gel pack (Freezit, Navagen, Bangalore) with surface temperature ≤5°C placed in contact (avoiding pressure) with the eye through closed lids for ten minutes.

The following primary outcome parameters were recorded sequentially:

1. Immediately after warming (or cooling) at time = 0 min: OST.
2. The intraocular pressure (IOP) was recorded as a secondary outcome parameter with the Pulsair Easy Eye air puff tonometer (Keeler instruments Ltd. SL4, 4AA, UK), as the median of three readings, taken within a minute of warming or cooling.
3. After 3, 6 and 9 min-Peak systolic and end diastolic CRA velocity.
4. After 10 min-OST and IOP were re-measured.

Data was analyzed using repeated measures ANOVA (RANOVA) and Tukey test for multiple comparisons with significance set at *P* < 0.05 (GraphPad Prism version 5.02); 95% confidence intervals are quoted.

## Results

We chose ten male and ten female healthy postgraduate-volunteers from the department. Their mean age was 26.4 years (SD 1.6). Baseline measures of pulse (μ ± SD: 81.6 ± 6.3 beats/min); systolic BP (μ ± SD: 119.1 ± 8.7 mmHg) and diastolic BP (μ ± SD: 77.1 ± 6.7 mmHg) were within normal range. The baseline values of OST, CRA PSV, EDV and RI and IOP are shown in Table 1.

**Table 1:** Summary of baseline variables of eyes

Variables	Minimum	Maximum	Mean ± S.D
Before warming (n = 20)			
OST* (°C)	32.00	36.40	34.5 ± 1.02
CRA PSV† (cm/s)	5.57	14.10	9.3 ± 2.33
CRA EDV‡ (cm/s)	2.66	7.98	4.6 ± 1.27
CRA RI§	0.29	0.67	0.5 ± 0.11
IOP (mmHg)	10.00	19.00	14.8 ± 2.34
Before cooling (n = 20)			
OST (°C)	33.00	36.20	34.5 ± 0.96
CRA PSV (cm/s)	6.02	14.60	9.7 ± 2.45
CRA EDV (cm/s)	3.19	7.04	4.7 ± 1.12
CRA RI	0.18	0.64	0.5 ± 0.13
IOP (mmHg)	11.00	20.50	15.0 ± 2.20

* OST - Ocular surface temperature;

† CRA PSV - Central retinal artery peak systolic velocity;

‡ EDV-End diastolic velocity;

§ RI - Resistive index

The OST increased to a mean of 36.4 ± 0.90°C immediately after warming. This reflects a significant mean rise of 1.96°C (95% CI 1.54 to 2.37). Ten minutes after cessation of warming, the OST had returned to near baseline values (μ: 34.4 ± 0.92°C). Compared to baseline, the changes in CRA parameters on warming are shown in Table 2.

**Table 2:** Changes in central retinal artery parameters after warming (n = 20)

Variables	CRA PSV* (cm/s) *P* < 0.0001	CRA EDV† (cm/s) *P* = 0.057	RI‡*P* = 0.28
Mean change			
At 3 min (μ ± S.D) and (95% CI)	1.21 ± 1.08 (0.5 to 1.91)	0.5 ± 1.19 (−0.08 to 1.08)	0.01 ± 0.11 (−0.07 to 0.09)
At 6 min (μ ± S.D) and (95% CI)	0.24 ± 1.45 (−0.46 to 0.94)	0.32 ± 1.23 (−0.25 to 0.90)	0.02 ± 0.14 (−0.10 to 0.06)
At 9 min (μ ± S.D) and (95% CI)	−0.75 ± 1.31 (−1.45 to -0.05)	0.01 ± 1.09 (−0.59 to 0.57)	0.04 ± 0.15 (−0.12 to 0.04)

* CRA PSV - Central retinal artery peak systolic velocity;

† EDV - End diastolic velocity;

‡ RI-Resistive index. The *P* value is for repeated measures ANOVA

The OST decreased to a mean of 31.7 ± 1.38°C immediately after cooling. This reflects a significant mean decrease of 2.81°C (95% CI −2.24 to −3.37). Ten minutes after cessation of cooling, the OST had returned to near baseline values (μ: 34.2 ± 0.93°C). Compared to baseline, the changes in CRA parameters are shown in Table 3.

**Table 3:** Changes in central retinal artery parameters after cooling (n = 20)

Variables	CRA PSV* (cm/s) *P* = 0.013	CRA EDV† (cm/s) *P* = 0.012	RI‡*P* = 0.81
Mean change			
At 3 min (μ ± S.D) and (95% CI)	−1.10 ± 2.26 (−2.05 to −0.15)	−0.80 ± 1.44 (−1.47 to −0.14)	0.02 ± 0.17 (−0.06 to 0.10)
At 6 min (μ ± S.D) and (95% CI)	−0.70 ± 1.44 (−1.65 to 0.25)	−0.64 ± 1.32 (−1.30 to 0.02)	0.03 ± 0.14 (−0.05 to 0.11)
At 9 min (μ ± S.D) and (95% CI)	−0.18 ± 1.25 (−1.13 to 0.77)	−0.37 ± 1.31 (−1.03 to 0.30)	0.02 ± 0.13 (−0.06 to 0.10)

* CRA PSV-Central retinal artery peak systolic velocity;

† EDV-End diastolic velocity;

‡ RI-Resistive index. The *P* value is for repeated measures ANOVA

At baseline, in 20 eyes, the IOP ranged from 10-19 mmHg [Table 1] and showed no significant change immediately after warming (*P* = 0.15; 95% CI −0.17to1.57). On cooling there was a significant drop in IOP by 3.32 mmHg (*P* < 0.0001; 95% CI: −4.30 to −2.35) The IOP had returned to pre-cooling levels ten minutes after cessation of cooling.

## Discussion

We studied the effect of warming (20 eyes) and (for the first time) cooling (20 eyes) on the CRA parameters of 20 healthy young volunteers.

Our baseline OST (34.5 ± 0.98°C) recorded with a thermo- anemometer were similar to those of Nagaoka (n = 10; 34.5 ± 0.2°C) measured with non-contact infrared radiation thermography.[6] Mori obtained baseline corneal OST (34.1 ± 1.2°C) no different from ours.[9] Efron, using wide-field color-coded infrared imaging device, demonstrated that temperatures at the limbus were on an average 0.45°C warmer than at the corneal center (34.3 ± 0.7°C).[10]

Our baseline CRA-PSV of 9.5 ± 2.4 cm/s was similar to that reported by numerous authors.[5,11–15] The mean CRA-PSV in these studies ranged from 8.3 to 12.5 cm/s. Dennis showed that CRA-PSV was faster when measured nearer the optic disc surface than at depth: Mean of 8.16 cm/s at 3.56 mm depth, and 13.89 cm/s at 1.76 mm.[16] The deeper value is comparable to our mean CRA-PSV of 9.5 cm/s obtained from a depth of 3 mm.

The baseline CRA-EDV of 4.7 ± 1.2 cm/s in our series appears higher than that of Guthoff[11] (n = 72; 3.1 ± 1.6 cm/s) and Ciulla[13] (n = 25; 1.9 ± 0.7 cm/s), but lower than that of Ashraf[17] (n = 29; 6.9 ± 2.1 cm/s). This could be on account of geographical variations, differences in people, machines (or manufacturers) and depth of measurements. Using Doppler, Ustymowics obtained EDV of 4.3 ± 1.2 cm/s at 2.1 ± 0.46 mm depth form the disc surface, and 3.6 ± 1.1 cm/s at 4.27 ± 0.9 mm; values quite akin to ours.[18] Our EDV is comparable to that obtained by Arai[15] (3.5 ± 1.6 cm/s) and Avanduk[14] (4.1 ± 1.9 cm/s).

The baseline CRA RI of 0.49± 0.12 compares well with Ashraf[17] (n = 29; 0.45 ± 0.28: Personal communication) and Avunduk[14] (n = 22; 0.54 ± 0.09), while being somewhat lower than values obtained by Arai[15] (n = 22; 0.72 ± 0.08) and Ciullla[13] (n = 25; 0.76 ± 0.06).

In our study, after ten minutes of warming, the OST rose significantly (μ rise 1.96°C; *P* < 0.0001; 95% CI: 1.54 to 2.37). In Nagaoka's study the OST rose by 3.3°C from a baseline of 34.5 ± 0.2°C to 37.8 ± 0.3°C on warming for ten minutes and like us, returned to baseline within ten minutes after cessation of warming.[6] Since the retino-choroidal tissue temperature is regulated to a lower level by the cooler anterior segment,[19] we concur with Nagaoka's reasoning that it is reasonable to assume that a rise in temperature anteriorly would have led to an increase in the posterior segment.[6] We had no means to measure retino-choroidal temperature.

Compared to baseline, after warming, the CRA-PSV was significantly higher at three minutes, near baseline values at six minutes and significantly lower at nine minutes [Table 2]. At the same times, the EDV and RI showed no significant change. In Nagaoka's study the blood velocity in the temporal retinal artery showed a transient but significant increase by 9.3 ± 1.9% at three minutes (and an increase of retinal blood flow of 14.2 ± 3.5%), returning to pre-warming levels by six. This compares favorably with our 13% increase in CRA-PSV at three minutes. It is our assumption that CRA velocity alterations faithfully reflect changes in its branches, including the retinal circulation. An increase in velocity without any alteration of RI would mean increased blood flow. One possible reason for the increased blood flow may be the enhanced retinal tissue energy demand on account of warming-associated metabolic changes. The significant fall, below baseline, of PSV at nine minutes is difficult to explain. We conjecture that it may merely be a case of rebound lowering once the heat stimulus was switched off. (Such a rebound phenomenon was not observed on chilling the eyes). Although not part of the protocol, additional readings three minutes later, showed no significant difference from the baseline.

The OST significantly decreased (95% CI: −2.24 to −3.37) on cooling but returned to near baseline values after ten minutes (95% CI: −0.93 to 0.21). Ortiz demonstrated a much greater lowering by 21.5°C using extremely cold air stream of −19°C for 40 min.[20] In another study, Katsimpris found that local hypothermia significantly reduces the temperature in the vitreous of rabbit.[8]

Both CRA-PSV and EDV significantly decreased on cooling at three minutes, but had returned to baseline values by six [Table 3]. No significant alteration occurred in the CRA RI. Despite an exhaustive literature search (PUBMED), we could not find any study which evaluated the effect of cooling on ocular blood flow. As opposed to warming, it is probable that cooling lowered the metabolic tissue demand, leading to a decrease in retinal blood flow. Moreover, hypothermia is known to cause arteriolar vasoconstriction and decreased blood flow in many tissues.[21] We had no means to measure the central retinal arterial diameters in our study.

It is important to consider changes in IOP on warming or cooling the eyes, since alterations of IOP may modify ocular perfusion. IOP has been reported to both decrease[7] and show no significant change on ocular warming.[22] In our analyses, there was no significant change in the IOP on warming. On the other hand, we observed a significant decrease in IOP immediately after cooling. This is somewhat more than the significant decrease reported by Ortiz[20] of 1.5 mmHg (attributed to a significant reduction of episcleral venous pressure) and 0.9 mmHg by Orgul.[23] To ensure that mere contact pressure of the gel pack was not leading to a decrease in IOP we repeated the measurement of IOP (on 10 eyes) at baseline, immediately after using a non-chilled gel pack for ten minutes and ten minutes later. We found no significant difference (RANOVA *P* = 0.7). The IOP rose to match the baseline values at ten minutes after cessation of cooling. Perhaps of greater importance is that despite a decrease in IOP the CRA, PSV and EDV were significantly lower on chilling. This suggests that cold exposure of eyes causes a significant reduction in ocular blood flow to the posterior segment and cryo-trauma to the eye would likely be an ischemic insult. Although Goldmann applanation tonometer is considered the gold standard, we preferred the air puff tonometer on account of it being quicker: And time was important for us, since the very nature of the study demanded numerous measurements in a limited time. Equally importantly, we were not studying IOP per se, but the change induced by our experimental intervention. For ‘change’ we felt the air puff tonometer would suffice.

Our study lacks in not having been able to co-evaluate the choroidal circulation and flow, largely on account of a lack of equipment. Subjective estimates of the choroidal blood flow although done, were considered to lack the objectivity to be seriously analyzed. This may be of greater import since choroidal perfusion abnormalities are reported in pathologies like age-related macular degeneration.[24,25]

Our study confirms that CRA blood flow significantly rises in response to ocular warming. In addition this is the first study to demonstrate a significant lowering of the CRA blood flow on cooling the eye. Cryo-injury to the eye has great importance for people who live in high altitudes or in extreme climatic zones like Ladakh (Tibet) and the Antarctic. The effect of cooling the eye on retinal blood flow sheds some light on this little researched aspect of ophthalmology. Additional research will help us understand the effects of heating and cooling modalities used in ocular therapy. Moreover, we can better predict their adverse effects.
